# *Hoplitolyda duolunica* gen. et sp. nov. (Insecta, Hymenoptera, Praesiricidae), the Hitherto Largest Sawfly from the Mesozoic of China

**DOI:** 10.1371/journal.pone.0062420

**Published:** 2013-05-03

**Authors:** Taiping Gao, Chungkun Shih, Alexandr P. Rasnitsyn, Dong Ren

**Affiliations:** 1 College of Life Sciences, Capital Normal University, Beijing, China; 2 Paleontological Institute, Russian Academy of Sciences, Moscow, Russia; 3 Department of Palaeontology, Natural History Museum, London, England; Ecole Normale Supérieure de Lyon, France

## Abstract

**Background:**

Large body size of an insect, in general, enhances its capability of predation, competition, and defense, resulting in better survivability and reproduction. Hymenopterans, most being phytophagous or parasitic, have a relatively small to medium body size, typically under 50.0 mm in body length.

**Principal Findings:**

Herein, we describe ***Hoplitolyda duolunica*** **gen. et sp. nov.**, assigned to Praesiricidae, from the Early Cretaceous Yixian Formation of China. This new species is the largest fossil hymenopteran hitherto with body estimated >55.0 mm long and wing span >92.0 mm. *H. duolunica* is, to our knowledge, the only sawfly with Sc present in the hind wing but not in the forewing. Its Rs1 and M1 meeting each other at 145° angle represents an intermediate in the transition from “Y” to “T” shapes. Even though *Hoplitolyda* differs significantly from all previously described genera in two subfamilies of Praesricidae, we leave the new genus unplaced in existing subfamilies, pending discovery of material with more taxonomic structure.

**Conclusions/Significance:**

*Hoplitolyda* has many unique and interesting characters which might have benefitted its competition, survival, and reproduction: large body size and head with robust and strong mandibles for defense and/or sexual selection, unique wing venation and setal arrangements for flight capability and mobility, dense hairs on body and legs for sensing and protection, etc. Considering the reported ferocious predators of feathered dinosaurs, pterosaurs, birds, and mammals coexisting in the same eco-system, *Hoplitolyda* is an interesting case of “survival of the fittest” in facing its evolutionary challenges.

## Introduction

Praesricidae is a small extinct sawfly family with four genera and six species described so far, all these species distributed in Central Asia. There are currently two subfamilies in the Praesiricidae: Praesiricinae Rasnitsyn, 1968 comprising *Praesirex* Rasnitsyn, 1968 and *Turgidontes* Rasnitsyn, 1990; and Rudisiriciinae Gao, Rasnitsyn, Ren & Shih, 2010 comprising *Aulidontes* Rasnitsyn, 1983 and *Rudisiricius* Gao, Rasnitsyn, Ren & Shih, 2010. Among them, only *Aulidontes* Rasnitysn, 1983 was reported from the Late Jurassic, while others were found from the Early Cretaceous [1]–[4]. *Xyelodontes* Rasnitsyn, 1983 was described in Praesiricidae as well, but currently it is transferred to Xyelydidae, which will be published in another paper.

Herein we report *Hoplitolyda duolunica* gen. et sp. nov., which was collected from Yixian Formation of Nanyingpan Village, the Sandaogou Township, Duolun County, Inner Mongolia, China. The age of this insect fauna is about 126 My, belonging to the famous Jehol biota [5], [6]. The fossil matrices are dark gray with darker insect imprints under low-angle lighting. The insect fauna found at this locality comprises many ephemeropteran nymphs of various stages, a lot of coptoclavids (Coleoptera), some mecopterans, trichopterans and dipterans, a few hymenopterans and a flea-like insect fossil, *Pseudopulex magnus* Gao, Shih & Ren, 2012, recently reported [7].

Records of giant hymenopterons are sparse. Shih et al. described a pelecinid, *Megapelecinus changi* Shih, Liu & Ren, 2010, with a body length of 50.9 mm from the Early Cretaceous of the Yixian Formation, Liaoning, China [8]. Archibald et al. reported a formicid, *Titanomyrma lubei* Archibald, Johnson, Mathewes & Greenwood, 2011, with a body length of approximately 51.0 mm from the latest Early Eocene of the Green River Formation, Wyoming, USA [9]. *Hoplitolyda duolunica* gen. et sp. nov. is the largest fossil hymenopteran hitherto with a very large body (estimated >55.0 mm long) and a broad wing span (>92.0 mm).

## Materials and Methods

### Material

We sorted two thousand hymenopteran fossils from Yixian Formation of China, but we only found one such sawfly. The specimen is housed in the Key Laboratory of Insect Evolution & Environmental Changes, College of Life Sciences, Capital Normal University, Beijing, China. No specific permits were required for the described field studies.

### Methods

The specimen was examined under a Leica MZ 16.5 dissecting microscope (Leica, Wetzlar, Germany). Line drawings were prepared with CorelDraw X6 graphics (Version 16.0.0.707) (Corel Corporation. USA) and Adobe Photoshop CS 6.0 (Version 13.0.1) (Adobe Systems Incorporated, USA) software. The photographs and magnified images of parts of the specimen were taken with a camera system connecting with the Leica MZ 16.5. Sometimes, we dropped ethanol (95%) on the surface of the specimen for much clearer photos. The wing venation nomenclature used in this paper is based on the interpretation of Huber and Sharkey, 1993 [10].

Venation abbreviations used in the text and figures: C, Costal; Sc, Subcostal; R, Radial; Cu, Cubital; A, Anal; Rs, Radial sector; M, Medial.

### Locality and Horizon

Nanyingpan Village is located in Sandaogou Township, Duolun County, Inner Mongolia, China. The age of the fossil-bearing beds in the Nanyingpan Village area is considered to be Early Cretaceous, Yixian Formation, around 126 Ma [11]–[13].

### Nomenclatural Acts

The electronic edition of this article conforms to the requirements of the amended International Code of Zoological Nomenclature, and hence the new names contained herein are available under the Code from the electronic edition of this article. This published work and the nomenclatural acts it contains have been registered in Zoobank, the online registration system for the ICZN. The Zoobank LSIDs (Life Science Identifiers) can be resolved and the associated information viewed through any standard web browser by appending the LSID to the prefix "Http://zoobank.org". The ISID for this publication is: urn:lsid:zoobank.org:pub:A0F09079-F194-47F7-820F-CC1FF5CAD718. The electronic edition of this work was published in a journal with and ISSN, and has been archived Anderson is available from the following digital repositories: PubMed Central and LOCKSS.

## Results

### Systematic palaeontology

Insecta Linnaeus, 1758
Order Hymenoptera Linnaeus, 1758
Superfamily Pamphilioidea Cameron, 1890
Family Praesiricidae Rasnitsyn, 1968
Subfamily indet.

### *Hoplitolyda* gen. nov

urn:lsid:zoobank.org:act:07BA2169-4010-4CC4-A12D-E0AB6E99A20

#### Type species

*Hoplitolyda duolunica* sp. nov.

#### Diagnosis

Large hymenopteron with head subcircular, widest at mandibular base. Mandible sickle-shaped, with single preapical tooth. Antenna with scape moderately long and 3^rd^ antennomere not disproportionally enlarged. Fore leg apparently resting on substrate with tibial apex and base of basitarsus flexed below tibia, with foretibial apical spur apparently lost. Forewing with Sc absent, R and pterostigma narrow, Rs1 reclined, meeting M1 at about 145°, RS+M very short, cells 1Rs and 1 M very long. Hind wing with Sc present.

#### Remarks

*Hoplitolyda* is assigned to Praesiricidae based on the combination of lost Sc in forewing, straight R, and trapezoidal mesopseudosternum widely reaching fore margin of mesoventropleuron (synapomorphies with Megalodontesidae, a living family known since the Early Cretaceous, from *Jibaissodes* Ren, Lu, Guo & Ji, 1995 [14], [15]) with bent M+Cu and simple flagellum. Unlike Praesiricidae, Megalodontesidae are apomorphic in having straight M+Cu and flagellum pectinate or flabellate. Its position in Pamphilioidea is confirmed: besides the general similarity in wing venation and in having long two-toothed mandibles, by the specific structure of the clypeus connected ventrally with the hypostomes and so with the mandibular and oral cavities separated. *Hoplitolyda* differs from other genera of Praesiricidae in its huge size, homonomous flagellum, head widest at mandibular base, forewing with comparatively thin R, narrow pterostigma, very long cells Rs and 1 M, hind wing Sc present, and in long pubescence on head and thorax. Some characters are possibly unique in all Symphyta, particularly the postocciput with transverse fore margin and narrow postoccipital bridge. Some others might be unique in all Hymenoptera: fore tarsus flexed far under tibia and with basitarsus much elongate, and M+Cu with a stub directed forward. That stub is symmetrical in both wings and so it looks like a normal character of the taxon. However, it is an unusual structure, a novelty that has no known homologue, and so also looks like an individual aberration. Only future finds might resolve this puzzle. One more possibly unique feature of the new genus is the putative absence of the antennal preening device including a modified hind (outer) apical spur and facing it an excision of the basitarsus. The only other member of Symphyta known to lack the apical foretibial spur is *Pachylota* Westwood, 1841 (Argidae) which has all the tibiae lacking apical spurs, unlike the present fossil.

As aforementioned, there are currently two subfamilies with four described genera. We add this fifth genus which differs much from all four other genera. *Hoplitolyda* could be easily segregated as a subfamily of its own. However, it is strange and excessive to have three subfamilies just for five genera. It might be wiser to leave the genus unplaced for now in the hope of getting more material that would make it possible to consider the taxonomic structure of the family in more detail.

#### *Etymology*

The name combines the Greek "Hoplit-" referring to "fully armed" and *Lyda* Fabricius, 1804, a junior synonym of *Pamphilius* Latreille, 1802 widely used to coin generic names in Pamphilioidea. Gender feminine.

### *Hoplitolyda duolunica* sp. nov. (Figs. 1, 2, 3)

urn:lsid:zoobank.org:act:68E7A450-6F4E-44B9-B2E6-0CBB0182A8E5

#### Holotype

No. CNU-HYM-ND-2011016, a well-preserved sawfly in ventral view, missing the terminal part of the abdomen. The specimen is deposited in the Key Laboratory of Insect Evolution & Enviromental Changes, College of Life Sciences, Capital Normal University, Beijing, China.

#### Description

**Figure 1:**
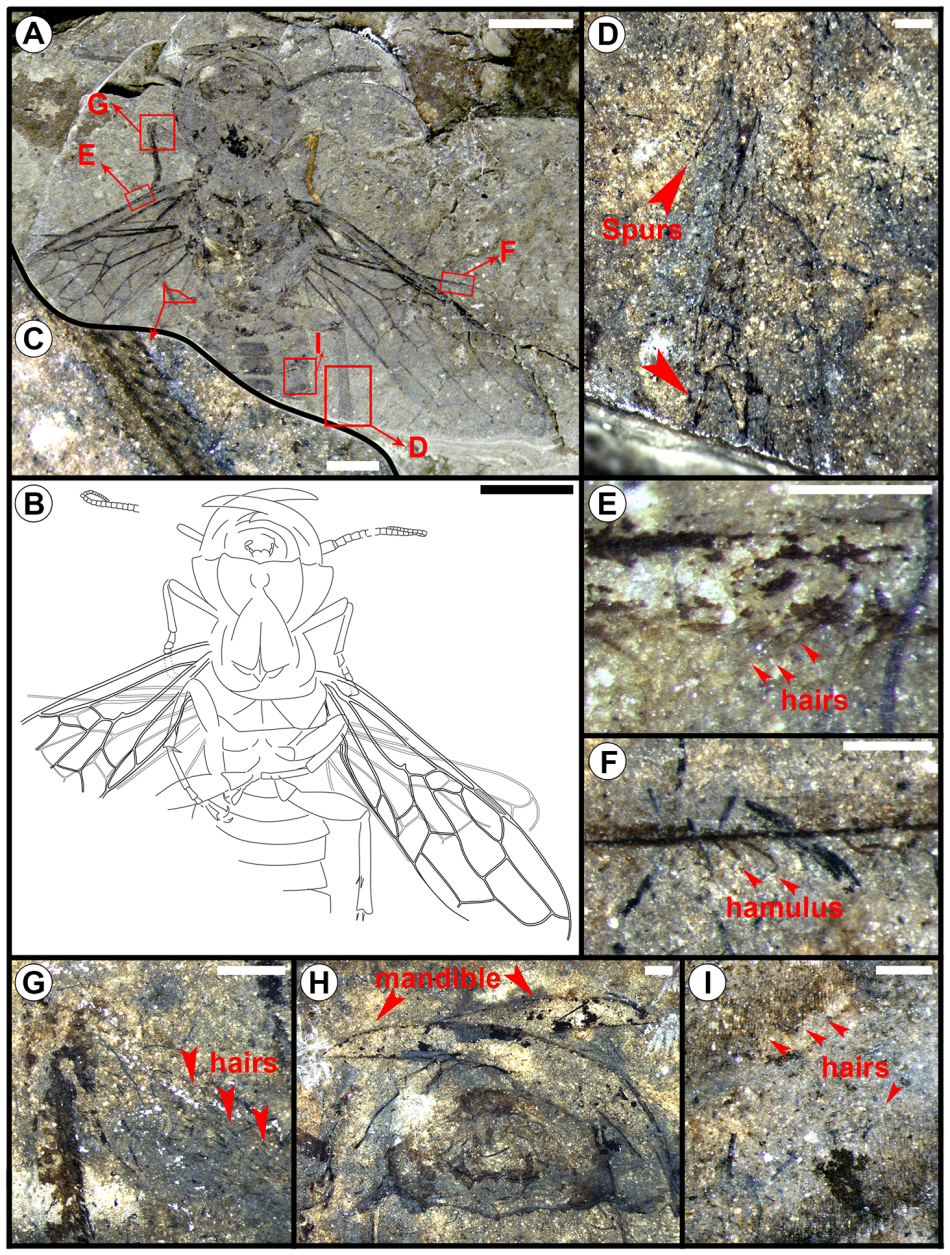
*Hoplitolyda duolunica* gen. et sp. nov., from the Early Cretaceous of China. A and B, photograph and line drawing; C, bristles on basitarsus of middle leg; D, spurs on hind leg; E, hairs below the vein C of forewing; F, hamuli on hind wing; G, hairs on tibia of fore leg; H, mandibles; I, hairs on abdomen. Scale Bars for A and B: 10.0 mm, for C to I: 0.5 mm.

Very large hymenopteron with a wing span >92.0 mm and body length as preserved about 46.0 mm (Figs. 1A, B). Missing abdominal segments 6 to 8 estimated with average length ∼3.5 mm each (by comparing ratios of abdominal segments to body length for species of *Rudisiricius* [4]), thus full body length estimated >55.0 mm. Body and legs covered with dense hairs. Surface sculpture not apparent. Color pattern not precisely known, head and thorax probably dark, abdominal segments (terga?) pale with dark median and posterolateral spots.

**Figure 2:**
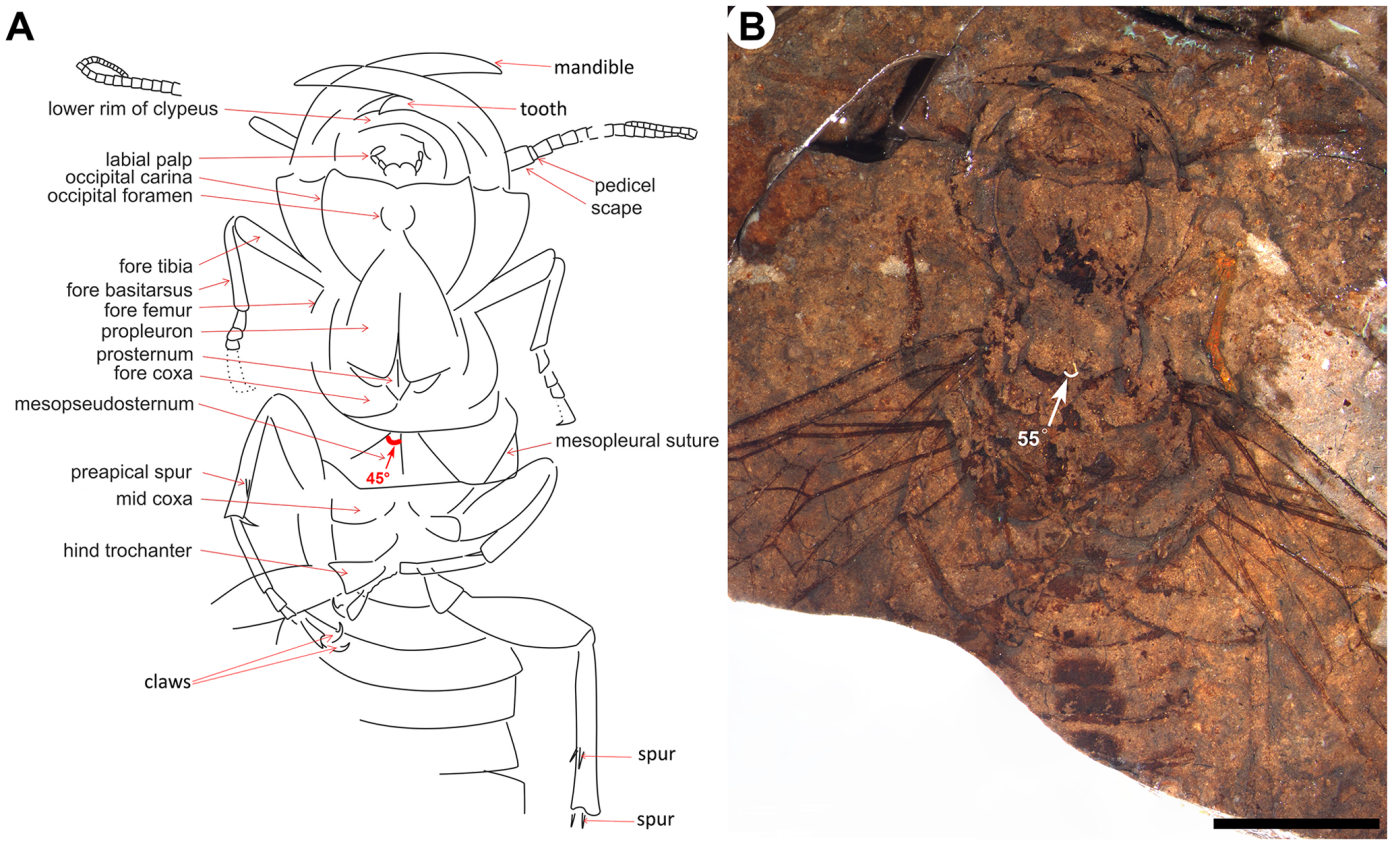
Detail structures of *Hoplitolyda duolunica* gen. et sp. nov. A, line drawing; B, photo under ethanol; Scale bar = 10.0 mm.

Head (Figs 1H, 2A, B) massive, width 15.4 mm, length without mandibles 9.3 mm, eyes unknown (evidently placed on invisible dorsal surface). Antenna somewhat longer than head width, with 27 visible antennomeres; scape more than twice as long as wide (incompletely preserved), possibly as long as 4–5 basal flagellomeres combined; pedicel very short; flagellomeres growing shorter and narrower toward apex, subquadrate or, subapical ones, elongate. Mandible (about 15.8 mm in length) long and sharp, reaching opposite side of head when closed, with long apical tooth and short, curved subapical one placed just beyond mandible midlength. Clypeus with wide semicircular lower rim joining lower head surface (apparently postocciput, but morphologically must be hypostome) laterally. Dorsal head surface not preserved, ventral one with complete occipital carina meeting hypostomal carina at mandibular base, with hypostome not distinctly visible: either not preserved or, rather, bent vertically, hypostomal carinae (visible only as fore margin of postocciput) directed strictly transversely, so forming almost straight line. Postocciput wide, delimited by semicircular occipital carina posteriorly and laterally, forming narrow postoccipital bridge with mesal boundary lost or not preserved. Maxilla not preserved; labium small, preserved as two apical lobes and three-segmented palp with two basal segments subequal and apical longest.

**Figure 3:**
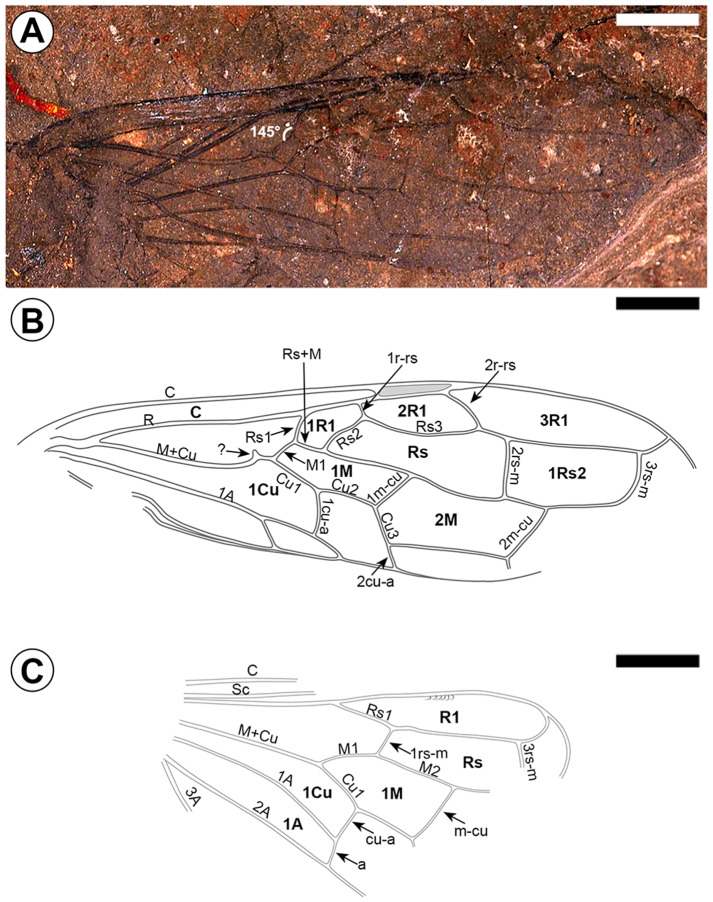
*Hoplitolyda duolunica* gen. et sp. nov., wings. A, photo of right wings; B, line drawing of forewing; C, Line drawing of hind wing; Scale bars = 5.0 mm.

Forewing (Figs 3A, B) 41.6 mm long, 11.5 mm wide, with delicate hairs anteriorly in costal space (Fig. 1E). Pterostigma narrow (5.4 mm long, 0.8 mm wide basally), fully sclerotized, wedge shaped. Sc absent. R straight (without distinct bend at base of Rs nor before it), not thicker than C. Rs1 (1.7 mm) slightly reclined toward wing base, almost aligned with M1, a little longer than M1 (1.6 mm), meeting M1 at about 145°. Rs+M (1.9 mm) a little longer than Rs1 or M1, reaching only 1/4 length of cell 1 M. Vein 1r-rs (∼0.9 mm) 1/3 length of 2r-rs (2.9 mm). Rs3 curved. M+Cu bent before vein M1, with a stub directed toward wing fore margin. Vein M lacking free apex. Cu nearly straight within cell 1 M, Cu1 (∼3.3 mm) slightly shorter than Cu2 (∼3.5 mm), but a little longer than 1cu-a (∼2.2 mm). Crossvein 1cu-a originating from Cu at midlength of cell 1 M. Cu3 aligned with 2cu-a. Crossvein 1 m-cu joining vein M at midlength of cell Rs, longer than M1 and as long as Cu3; 2 m-cu oblique, joining cell 1Rs2 at about 2/5 of its length. Crossvein 2rs-m well beyond 2r-rs; 3rs-m near apex of cell 3R1. Anal veins ordinary. Cell 1R1 very short; cells Rs and 1 M very long. Cell 1 M with fore and hind margins almost straight. Hind wing (Figs 3A, C) with at least 10 hamuli near midlength of cell R1 (Fig. 1F); cell R1 widely rounded apically. Vein Sc well developed. Rs1 slightly longer than M1. Crossvein 1rs-m distant from bases of both Rs and M, half as long as 1 M; 3rs-m near apex of cell R1. Crossvein m-cu long, joining M2 near midlength of cell Rs. Vein M+Cu and 2A nearly straight; 1A curved near midlength. Vein 3A present. Crossvein cu-a before midlength of M. Cells 1Cu and 1A truncated before midlength of cell 1 M. Cell 1Rs2 slightly longer than cell 1 M.

Thorax (Fig. 2) slightly wider than head, covered with dense hairs of varying length. Propleura long, about 1/2 width of head, but nearly same height, left and right part of propleuron displaying a 55° angle (Fig. 2B). Prosternum narrow diamond-like shaped, half inserted between propleura. Mesoventropleuron short; mesopseudosternum wide trapezoidal in form, wide on its fore margin. Pseudosternal sulci ca. 45° to fore and hind margins of ventropleuron (Fig. 2A).

Coxae and femora short. Fore leg (Fig. 2) with femur narrow, curved, only slightly thicker than tibia; tibia narrow, covered with thick hairs (Fig. 1G), no apical spurs preserved; basitarsus long and narrow (∼5.3 mm), shorter than tibia (∼7.2 mm), slightly widened apically, slightly shorter than remaining tarsomeres combined, with many thick bristles on flexor surface (Fig. 1G) (like at least mid basitarsus (Fig. 1C) and hind tibia (Fig. 1D)); tarsomere 2 only slightly elongate, 3 and 4 subquadrate, 5 (poorly preserved) longer than two preceding combined, with strong claw. Both fore basitarsi preserved flexed under tibia as if fore leg rests on substrate on tibial apex and basitarsal base. Mid and hind legs ordinary; mid and hind trochanters triangular, slightly longer than wide; femora short, fusiform, hind one thicker. Mid tibia thin, as long as femur, with apical and preapical spurs rather short; tarsus much longer than tibia, with basitarsus about as long as remaining tarsomeres combined, tarsomeres 2 and 3 elongate, 4 ill-preserved, 5 longer than 2, with big claws bearing prominent basal lobe, otherwise simple. Hind tibia about as long as femur with trochanter, with apical and preapical spurs short; tarsus not preserved.

Abdomen about as wide as head and thorax, with segments 2–7 partly visible, segments short, covered with dense hairs laterally (Fig. 1I).

#### Occurrence

Nanyingpan Village, Sandaogou Township, Duolun County, Inner Mongolia, China. Yixian Formation, Early Cretaceous.

#### Etymology

The specific epithet "*duolunica*" from "duolun" referring to the locality of the fossil.

## Conclusions

Giant ancient winged insects have been reported from Late Paleozoic strata, but insect body size decreased in the Mesozoic and reached the same size as extant ones, possibly due to reduction of atmospheric oxygen level [16]–[18] and/or the emergence of efficient insect-eating vertebrates [19]. Other factors affecting insect body size are environmental temperature, food availability, isolation (so called “island rule”), eco-systems, or sexual selection [20], [21]. Large body size of an insect, in general, might enhance its capability of predation, competition and defense resulting in better survivability and reproduction, although opposite-directed pressure is quite common as well. Gigantism of *H. duolunica* gen. et sp. nov., as contrasted with other coeval sawflies, might have been caused by sexual selection or just by niche segregation within their eco-system to avoid competition.

Male insects, especially of large body size, often have elaborate and conspicuous ornaments or weapons of exaggerated proportions [22]. The size and conspicuousness of these structures make them likely candidates for intraspecific signals, used either by males to assess the competitive status of rival males, or by females to assess the relative suitability of potential mates [22], [23]. *Hoplitolyda duolunica* gen. et sp. nov. has a large head with robust and exaggerated mandibles, a common character of the praesiricid Rudisiriciinae and the *Ferganolyda* Rasnitsyn, 1983 males of the family Xyelydidae [24]. Mandibles of *H. duolunica* sp. nov., robust and huge with sufficient support from the big head (Fig. 2B), could be stretched at a wide angle (Fig. 1H). Thus, these mandibles might have been used as weapons for defense, the same as many extant pamphilioids (Family Pamphiliidae). It is interesting to note that when sexed, all the fossil pamphilioids with exaggerated robust mandibles are males; it is likely that the huge mandibles were also used as a sexual display in deterring other rival males and/or attracting females. On the other hand, the extant Pamphilioidea (Pamphiliidae and Megalodontesidae) equally have a big head with long and powerful mandibles but display no real sexual dimorphism in that respect. Head and mandible hypertrophy in *Hoplitolyda* was hardly connected with its feeding habits, for its weakly developed labium might even indicate aphagy.

Praesiricidae is a small family of Symphyta, now with only seven described species in five genera. A majority of the species is confined to the Early Cretaceous (Berriasian through Aptian), while only *Aulidontes mandibulatus* Rasnitsyn, 1983 is described from the Late Jurassic. Despite the narrow age period, the family displays a wide range of morphological variations in body size, head, antenna, mandible, leg and wing venation. Size variation is great, as shown by the most indicative forewing length, ranging from 8.5 mm in *Aulidontes mandibulatus* to 41.6 mm in *H. duolunica* sp. nov., which is the largest known sawfly. Antennal variation is shown by scape huge (as long as the head) and basiflagellar antennomeres disproportionally long (*Rudisiricius*, *Aulidontes*), in contrast to only basiflagellar antennomere much elongate (*Praesirex*, *Turgidontes*) or no antennomere disproportional (*Hoplitolyda*). The head can be almost normal (*Praesirex*, *Turgidontes*, *Aulidontes*) or very large with huge mandibles (*Rudisiricius* and particularly *Hoplitolyda*), even though the head is never as monstrous as that of *Ferganolyda* in Xyelydidae (Rasnitsyn et al., 2006). Legs vary from apparently normal (most genera, even though their legs are never described in sufficient detail) to uniquely modified in *Hoplitolyda* to use the fore-tibial apex along with the base of the down-flexed basitarsus as a resting point for the fore leg, while the antennal preening apparatus is apparently lost. Wing venation varies in these characters: size of pterostigma (narrow in *Hoplitolyda* vs. moderately big in all other genera); Rs1 moderately long and proclival (*Aulidontes*), vertical (*Rudisiricius*) or reclival (*Hoplitolyda*), or else short and reclival (*Praesirex*) or proclival (*Turgidontes*); Rs+M almost as long as cell 1 M (*Praesirex, Turgidontes*), half as long (*Rudisiricius*, *Aulidontes*), or very short (*Hoplitolyda*); cell 1 M small (*Rudisiricius*, *Aulidontes*), long and high (*Praesirex*, *Turgidontes*), or low but very long (*Hoplitolyda*); and angle of Rs1 meeting M1 from 93° (*Aulidontes*) to 180° (*Rudisiricius*).

**Figure 4:**
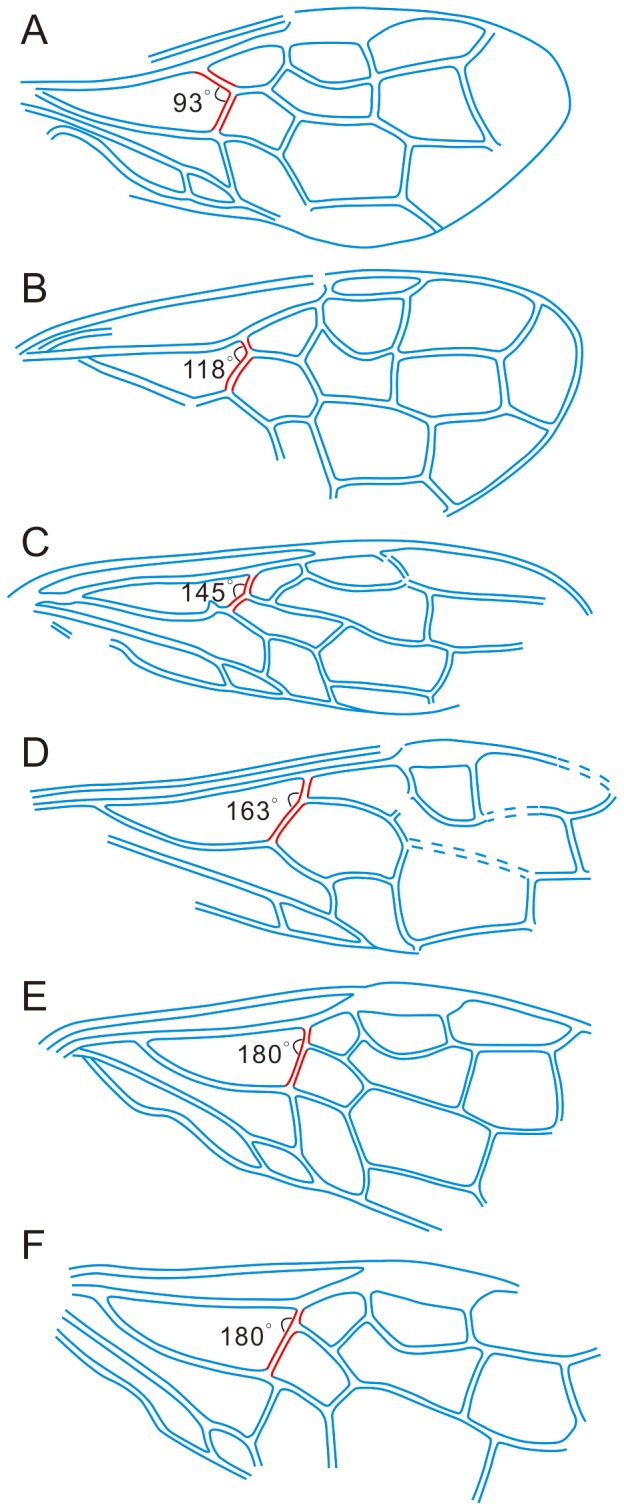
Transition of wing venation from “Y” shape to “T” shape in Pamphilioidea. A, *Aulidontes mandibulatus* Rasnitsyn, 1983; B, *Juralyda udensis* Rasnitsyn, 1977; C, *Hoplitolyda duolunica* gen. et sp. nov.; D, *Praesirex hirtus* Rasnitsyn, 1968; E, *Rudisiricius crassinodus* Gao, Rasnitsyn, Ren & Shih, 2010; F, *Rudisiricius celsus* Gao, Rasnitsyn, Ren & Shih, 2010.

An evolutionary trend is revealed by the sections of Rs and M in the course of formation of the so-called basal vein (red in Fig. 4) as represented by a series of sawfly fossils. Plesiomorphically, as shown by most Xyelidae, Rs1 and M1 meet each other at an acute or right angle (forming a “Y” shape together with Rs+M), and this character state is apparently lost in all Pamphilioidea, mainly as a byproduct of shortening of Rs1 (Fig. 4). The most basal case can be found in *A. mandibulatus* (Fig. 4A) [3], while the other fossils show a transition (Figs 4B–D) [1], [25] toward a perfectly linear alignment of Rs1 and M1 (Figs 4E, F) (forming a “T” shape together with Rs+M) [4], which is the (true) basal vein present in a majority of Hymenoptera Apocrita [8], [26]. The new genus represents an intermediate position in the transition from “Y” to “T” shapes. The well-developed basal vein is probably important aerodynamically. In general, wing structures suggest that *H. duolunica* sp. nov. was a good flyer, although not an excellent one.

The wing venation of *H. duolunica* sp. nov. is unique in that it has no Sc in the forewing in contrast to the hind wing. The most basal sawflies possessed Sc in both wing pairs in contrast to some derived taxa and to all higher Hymenoptera which lost Sc completely [2], [27]. As a transition, we can observe forewing Sc retained as a longitudinal vein, and hind wing lacking any Sc (in majority of Xyelotomidae and Xyelydidae, in living and some extinct Siricidae, and in Protosiricidae). However, *H. duolunica* sp. nov. is, to our knowledge, the only example when Sc is present in the hind-and not in the forewing. The new species is also unique in having a forward stub on its M+Cu, but, as afore-mentioned, more material is needed to be certain if it is a novelty of evolutionary meaning, or simply an individual malformation.

In summary, *Hoplitolyda* gen. nov. has a large body size and exaggerated mandibles possibly for defense and/or sexual selection, its unique wing venation and setal arrangements to enhance flight, dense hairs on body and legs for sensing and protection. Considering the reported ferocious predators, feathered dinosaurs, pterosaurs, birds, and mammals [28]–[31], coexisting in the same eco-system, *Hoplitolyda* gen. nov. has demonstrated an interesting example of “survival of the fittest” in facing its evolutionary challenges.
